# Direct and indirect effects of copepod grazers on community structure

**DOI:** 10.1093/plankt/fbae047

**Published:** 2024-09-16

**Authors:** Kristie Rigby, Elisa Berdalet, Carina Berglund, Fabian Roger, Michael Steinke, Mahasweta Saha, Wiebke Grebner, Emily Brown, Uwe John, Lars Gamfeldt, Patrick Fink, Fredrick Berggren, Erik Selander

**Affiliations:** Department of Marine Sciences, University of Gothenburg, Carl Skottsbergs gata 22B, Gothenburg 41319, Sweden; Department of Marine Biology and Oceanography, Institute of Marine Sciences (ICM-CSIC), Passeig Marítim de la Barceloneta 37-49, Barcelona 08003, Spain; Department of Marine Sciences, University of Gothenburg, Carl Skottsbergs gata 22B, Gothenburg 41319, Sweden; Department of Marine Sciences, University of Gothenburg, Carl Skottsbergs gata 22B, Gothenburg 41319, Sweden; School of Life Sciences, University of Essex, Wivenhoe Park, Colchester CO4 3SQ, UK; Marine Ecology and Biodiversity, Plymouth Marine Laboratory, Prospect Place, Plymouth, Devon PL1 3DH, UK; Department of Marine Sciences, University of Gothenburg, Carl Skottsbergs gata 22B, Gothenburg 41319, Sweden; School of Biological Sciences, Georgia Institute of Technology, Atlanta, GA 30332-0230, USA; Department of Ecological Chemistry, Alfred-Wegener-Institute, Helmholtz Center for Polar and Marine Research, Am Handelshafen 12, Bremerhaven 27570, Germany; Helmholtz Institute for Functional Marine Biodiversity, University of Oldenburg, Ammerländer Heerstraße 231, Oldenburg 26129, Germany; Department of Marine Sciences, University of Gothenburg, Carl Skottsbergs gata 22B, Gothenburg 41319, Sweden; UFZ Department River Ecology and Department Aquatic Ecosystem Analysis, Helmholtz Centre for Environmental Research, Brückstr. 3a, Magdeburg 39114, Germany; Department of Marine Sciences, University of Gothenburg, Carl Skottsbergs gata 22B, Gothenburg 41319, Sweden; Department of Marine Sciences, University of Gothenburg, Carl Skottsbergs gata 22B, Gothenburg 41319, Sweden

**Keywords:** ecosystem function and services, multi-trophic interactions, plant–herbivore interactions, algae, indirect effects, direct effects, structuring effects, chemically mediated interactions

## Abstract

Ecological theory and empirical research show that both direct lethal effects and indirect non-lethal effects can structure the composition of communities. While the direct effects of grazers on marine phytoplankton communities are well studied, their indirect effects are still poorly understood. Direct and indirect effects are inherently difficult to disentangle in plankton food webs. In this study we evaluate the indirect effects of copepod grazers on community function and structure using isolated chemical alarm signals, copepodamides. We expose intact summer and spring communities to direct grazing from copepods, or to chemical alarm cues without the presence of grazers in controlled experiments. The effects of direct grazing on ecosystem function were moderate in both experiments as indicated by levels of chlorophyll and primary production. Indirect and direct effects resulted in changes in the composition of both the eukaryote and prokaryote communities as shown by metabarcoding of 18S and 16S rRNA. Size structure analysis suggests that direct grazing and copepodamide exposure both favoured smaller organisms (< 10–15 μm) corroborating the size-structuring effect of copepod grazers. We conclude that the well-established effect of copepods on phytoplankton communities results from a combination of direct and indirect effects. This is a first attempt to isolate indirect effects of copepods on community structure and the results suggest that a full mechanistic understanding of the structuring effect of copepods will require insights to both direct and indirect effects of consumers as demonstrated for other ecosystems components.

## INTRODUCTION

Consumers induce defensive traits in prey organisms such as cryptic behavior or morphologically defended phenotypes (Tollrian and Harvell, 1999). The induced traits alter predator–prey interactions and drive cascading effects in food-web dynamics. Indirect trait-mediated effects like this are common in most ecosystems and may rival or even exceed the direct effects of predation (Preisser *et al.*, 2005; Suraci *et al.*, 2016). For example, the presence of piscivorous fish may result in altered habitat use of planktivorous fish in lakes. The spatial redistribution of planktivorous fish in turn releases zooplankton from predation leading to several-fold increase in zooplankton density (Turner and Mittelbach, 1990). Marine plankton are less studied and are hard to assess primarily due to their higher temporal and spatial variability as well as difficulties in tracking the variety of the involved microbial components. The comparably few studies on cascading effects in marine plankton show inconsistent results (Shurin *et al.*, 2002). Predation rates are, however, higher in marine plankton than in most systems: around 80% of the marine primary production is typically consumed by grazers (Calbet, 2001; Calbet and Landry, 2004). In addition, many plankton organisms respond to chemical alarm cues from consumers by expressing defensive traits such as cryptic behaviors (Long *et al.*, 2007; Selander *et al.*, 2011; Bergkvist *et al.*, 2012), onset of diel feeding rhythms (Arias *et al.*, 2021), life history changes (Toth *et al.*, 2004), colony size alterations (Bjærke *et al.*, 2015) or spine formation (Van Donk *et al.*, 2011). Cascading effects have also been implicated upon introduction of invasive consumers such as the comb jellyfish *Mnemiopsis leidyi,* which efficiently suppresses mesozooplankton grazers in invaded habitats with cascading effects on phytoplankton production and composition (Tiselius and Møller, 2017). At the microscale, however, it is challenging to separate direct effects of consumers from indirect effects. The individual actors cannot be easily manipulated and to what extent effects are driven by direct consumption or trait-mediated indirect effects such as intimidation of prey organisms remains an open question.

Here, we use chemical alarm signals from copepods to isolate direct from indirect effects leaving apart the effects of nutrient regeneration due to copepod grazing. Copepods were targeted due to their role as key intermediate consumer group in marine food webs (Turner, 2004), being the principal link from marine primary production to higher trophic levels. In addition to consuming prey, they imbue seawater with a unique bouquet of polar lipids, so called copepodamides (Selander *et al.*, 2015). Copepodamides act as a general alarm signal inducing defensive traits in prey organisms such as diatoms, dinoflagellates and ciliates. Responding organisms increase their bioluminescence, toxin production, decreased colony size or initiate diel feeding rhythms (Selander *et al.*, 2015; Lindström *et al.*, 2017; Selander *et al.*, 2019; Arias *et al.*, 2021; Rigby and Selander, 2021). Moreover, copepodamides reach bioactive levels in nature and have been shown to drive trait-mediated effects in experiments with artificially assembled communities (Prevett *et al.*, 2019; Selander *et al.*, 2019). However, the effect of copepodamides on community structure has not yet been tested experimentally in more complex settings such as natural communities. Grazer-induced phytoplankton phenotypes can be fully or partly reproduced by copepodamide exposure alone (Grebner *et al.*, 2019; Selander *et al.*, 2019). Thus, copepodamides can be used to mimic predator presence without the presence of actively feeding copepods.

The structuring effects of copepods have been described by Stibor and colleagues (Stibor *et al.*, 2004). Copepods are omnivorous and feed on a variety of prey organisms. Larger and faster-swimming prey are cleared at higher rates than smaller and slower organisms (Berggreen *et al.*, 1988; Selander *et al.*, 2011). Thus, grazing copepods can promote the presence of smaller life forms (e.g. Ryther and Sanders, 1980; Calbet and Saiz, 2005). Additionally, the removal of microzooplankton, such as ciliates and heterotrophic dinoflagellates, further reduces predation pressure on smaller cells. The structuring effect of copepods is likely a combination of direct grazing and indirect effects such as intimidation of microzooplankton grazers, e.g. onset of diel feeding rhythms or reduced swimming velocity (Arias *et al.*, 2021), or induced break-up of colonies into smaller units (Selander *et al.*, 2019; Rigby and Selander, 2021). Hence, we hypothesize that the addition of copepods, or the chemical alarm signals from copepods alone, will both favour smaller and more defended life forms (Selander *et al.*, 2019). The direct effects include selective removal of larger cells and microzooplankton grazers, which both will lead to increased relative abundance of smaller cells, which should in theory also include heterotrophic nanoflagellates that leads to increased grazing on the prokaryote community. Copepodamides can be predicted to drive trait-mediated indirect effects enhancing the direct effects. We predict the effect to be larger during the spring bloom compared to during the summer. In summer, plankton communities in temperate waters are likely already adapted to high grazing rates from copepods and the community response to copepod grazing or copepodamides has already been manifested.

## MATERIALS AND METHODS

### Study sites and sampling

Experiments were performed off the Swedish west coast, north-east Atlantic, which is characterized by temperate seasonality. Production is low in winter and increases sharply during the spring bloom in February–March. The spring bloom is characterized by chain-forming diatoms (Tiselius and Kuylenstierna, 1996) followed by an increase in microzooplankton grazers and subsequently also copepod biomass, which is typically around 20 times higher in summer than winter (Kiørboe and Nielsen, 1994; Nielsen and Kiørboe, 1994). A community dominated by smaller flagellated cells develops in the more oligotrophic summer conditions. We used the experimental facilities at Tjärnö Marine Laboratory (summer experiment May 2016), and Kristineberg Center (spring bloom experiment February 2017). Seawater was retrieved from the depth of the chlorophyll maximum using water collectors of the Niskin type in summer (N58.52‘69.4, E11.05’40.5), and spring (N58.15′816, E11.27′44.2). Copepods were collected using a vertical tow with a plankton net (WP2 200 μm mesh size, 100 m depth) and a 90 μm mesh at a depth of 20 m. Contents from the 90 μm net were further filtered through a 65 μm net and all nets were equipped with a non-filtering cod end. The net hauls were immediately diluted with filtered seawater and transported back to the lab. The summer campaign was held in the Koster fjord region of the Skagerrak that hosts a large population of *Calanus finmarchicus* (Båmstedt *et al.*, 1990). The first campaign in Summer 2016 was part of a large collaborative workshop, while the spring campaign was a follow-up campaign on a smaller scale which included fewer parameters (Table 1).

**Table 1:** Measured parameters for the Summer 2016 and Spring 2017 campaigns

Analysis	Summer 2016	Spring 2017
Primary production	Yes	No
Bacterial production	Yes	No
Bacterial counts	Yes	No
Chlorophyll analysis	Yes	No
DMSP quantification	Yes	No
Copepodamides	Yes	Yes
Microscopy analysis	Yes	Yes
Size distribution	Yes	Yes
Metabarcoding	Yes	Yes

### Copepodamide isolation

Copepodamides were isolated from commercially available freeze dried *C. finmarchicus* (Calanus AS, Tromsø, Norway) as described in Selander *et al.* (2015). A mixture of copepodamides representing the natural blend from *C. finmarchicus,* which are also one of the dominating species in the area (Båmstedt, 1988), were used in experiments. Details on general and species-specific copepodamide compositions are available in Selander *et al.* (2019) and Grebner *et al.* (2019), respectively.

### *Experimental* setup

**Fig. 1:**
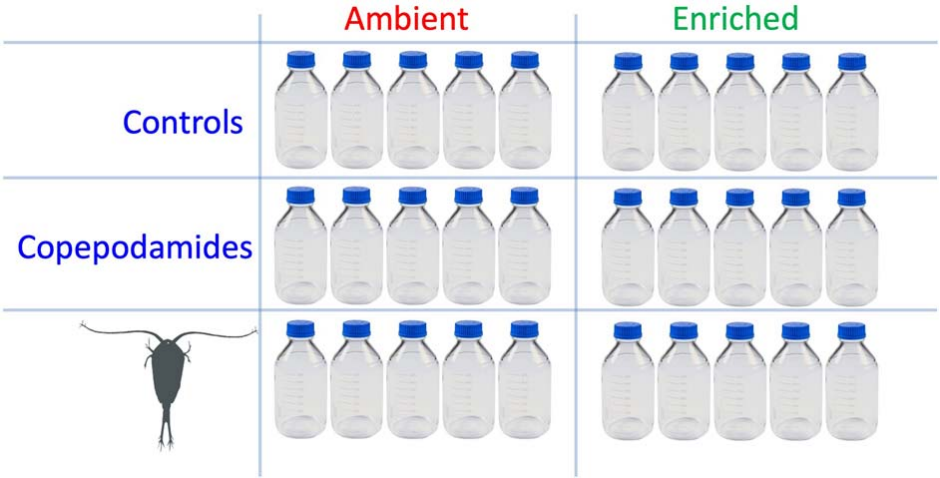
Schematic outline of the factorial design with three levels of grazing, control (no addition), copepodamide addition and direct grazing by copepods. The setup was repeated with enriched flasks to assess the importance of the fertilizing effect of copepods. 30 bottles in total with five replicates per treatment, six treatments in total.

The experiments were carried out in 1140 mL glass bottles (Fisher Scientific), fitted with Teflon-lined silicon inserts in the screw caps to enable bubble-free sealing. The summer community was gently pre-filtered through a submerged plankton mesh (65 μm) to remove naturally occurring copepods. The spring bloom community was not prefiltered due to negligible densities of copepods and the presence of chain-forming diatoms large enough to be retained by the mesh. The water was divided into 30 bottles (Fig. 1) with five replicates and six treatment combinations. Ten had been pre-coated with 50 nmol copepodamides dissolved in 80 μL methanol and 10 controls with 80 μL methanol alone. The methanol was evaporated before the seawater was added. Re-coating of the bottles was performed every other day over the course of the experiment. Administered like this, the average effective concentration (resulting from release from the bottle wall and degradation in the water) averages around 1% of the nominal content (Selander *et al.*, 2019). The effective concentration of copepodamides was measured at the end of the experiment. The remaining 10 bottles received copepods from the sample taken at the sample site and in approximately the same relative abundance (10 *Acartia* sp. and 1 *Calanus* sp. per bottle for the summer campaign, three *Temora longicornis,* two *Paracalanus parvus,* three *Acartia* sp. and four *Centropages* sp late stage copepodites or adults for the spring campaign). Five replicates from each treatment were supplemented with nutrients, nitrate (NO_3_^−^, 220 μM), phosphate (PO_4_^3−^, 9 μM) and silicate (Si, 26.6 μM). Monitoring stations closest to sampling sites (<500 m, within 2 weeks) report NO_3_^−^ 0.14 μM, PO_4_^3−^ 0.15 μM and SiO 2 μM for the spring campaign, NO_3_^−^ 0.1 μM, PO_4_^3−^ 0.04 μM and Si 0.1 μM for the summer. Bottles were placed on a plankton wheel with a rotation of 0.5 rpm at a 16:8 light:dark cycle (100 μmol photons m^−2^ s^−1^) at 4°C or 16°C for 5 days (spring and summer campaigns, respectively).

### Analysis

#### Primary production

Primary production was measured by isotope incorporation experiments (Nielsen and Bresta, 1984). A well-mixed 10 mL subsample was transferred to 25 mL scintillation vials and received 10 μCi H^14^CO_3_. The vials were incubated standing in the same conditions as the main experiment for 4 h. Uptake of labeled carbonates was terminated by addition of 150 μL formaldehyde. Remaining inorganic H^14^CO_3_ was degassed as ^14^CO_2_ by purging the solution with air after acidification with three drops of concentrated hydrochloric acid. Samples received scintillation cocktail (Instagel, Perkin Elmer) and were read on a scintillation counter (Beckman LS6000).

#### Bacterial production

We evaluated the bacterial production and counts to investigate potential cascading effects on food web structure either from direct consumption or indirect effects (Preisser *et al.*, 2005). 1 mL of the bottle content was transferred to Eppendorf tubes and supplemented with 57 nmol tritium-labeled thymidine, corresponding to 1 mCi mL^−1^. The samples were incubated for 1 h in darkness. The exact time was noted, and the incorporation was stopped by adding 150 μL formaldehyde. Controls (n = 3) were treated the same way but received 150 μL of formaldehyde prior to thymidine addition. The tubes received 89 μL 100% ice cold TCA and were centrifuged at 13 000 rpm for 10 min. The supernatant was removed, and the pellet resolved in 1.5 mL ice-cold 5% TCA (aq) and centrifugation repeated. Finally, the pellet was resolved in 1.5 mL 80% ethanol before centrifuging again and removing the supernatant. The sample received scintillation fluid and was analyzed in a scintillation counter as above.

#### Bacterial counts

Samples (5 mL) were fixed with 4% v/v Formaldehyde at the end of the experiment and stored at −20°C. Per sample 1 mL was mixed with 5 μL SYBR Green (1:100 from stock 10uM) and incubated in the dark for 15 min. The samples were supplemented with 15 or 5 μL bead solution (CountBright Absolute Counting Beads for flow cytometry) before analysis with BD FACSCalibur Flow Cytometer.

#### Chlorophyll analysis

During the summer campaign, chlorophyll samples were taken at the beginning and end of the incubation. Water from each replicate was filtered through a 25 mm GF/F filter (Whatman). Filters were then wrapped in aluminum foil and stored at −20°C. The GF/F filters were extracted in 20 mL of 96% Ethanol for 24 h at room temperature in darkness before analysis on a fluorometer (Turner designs, AU-10) as described in (Strickland & Parsons, 1972). A blank sample readout (96% ethanol) was subtracted from all measurements.

#### Quantification of DMSP

Dimethylsulfoniopropionate (DMSP) is an infochemical that mediates species interactions including grazing, an osmolyte in many phytoplankton and the precursor for dimethyl sulfide (DMS) (Strom *et al.*, 2003). DMSP was quantified using gas chromatography with flame-photometric detection after alkaline hydrolysis to DMS. On day 3 of the incubation, 30 to 50 mL of water sample from the experiment were gravity-filtered onto 25 mm GF/F filters (Whatman) in analytical triplicates. Filters were folded in half and placed into glass vials (volume of 4.92 mL) with 3 mL of 0.5 M NaOH, and immediately closed gas tightly using screw caps with Teflon-coated silicon septa. Headspace gas (200 μL) from the vials (n = 90 including filter blanks) was injected into a gas chromatograph (Shimadzu GC-2014) equipped with a wide-bore column (Agilent HP-1, 23 m × 0.53 mm × 5 μm). Methodological details for the gas chromatograph settings were as reported in Franchini and Steinke (2016). The limit of detection for DMSP in seawater was < 7.7 nM based on smallest concentration used in the calibration.

#### Copepodamides

Copepodamides were extracted from 200 mL of seawater using 100 mg ABN solid-phase extraction columns, a functionalized polystyrene divinylbenzene polymer that retains copepodamides from seawater. The columns were washed with one column volume of milliQ water to desalt. Copepodamides were eluted in two times 1.5 mL methanol separated by a 30 s soak step to maximize yield. The eluate was evaporated at 40°C under a stream of nitrogen and resolved in 70 μL methanol before analysis on triple quadrupole LC–MS (see Selander *et al.*, 2015 for details).

#### Phytoplankton microscopic analysis

100 mL samples were preserved in acidic Lugol solution and stored at 4°C. Analysis was completed using Utermöhl’s inverted microscope technique (Utermöhl, 1931). Chambers were filled with 50 mL of the preserved sample from each replicate and left to settle overnight, whereafter plankton composition was determined under an inverted light microscope.

#### Size distribution

Coulter counters (summer campaign: Beckman Multisizer 3; spring campaign: Elzone) were used to determine size distribution of cells between 4 and 25 μm equivalent diameter size. A well-mixed sample was taken from each bottle. The size distribution was determined in fresh, un-preserved samples. At least 1 mL was analyzed for each replicate.

#### Metabarcoding

The prokaryote and eukaryote community composition were analyzed through the metabarcoding of 16S rRNA and 18S rRNA genes respectively. The metabarcoding provides good qualitative resolution but does not translate to direct quantitative differences because of the highly variable copy numbers in different taxa (Martin *et al.*, 2022). However, for assessing changes in community composition, copy number variation is less problematic because of its proportional influence on overall dissimilarity calculations, particularly with standardized data. Additionally, an alternative analysis using rarefied data and a frequency transformation (effectively removing any abundance differences between ASVs) did not qualitatively alter the results or conclusions. For DNA extraction, 250 mL samples were filtered through sterivex filters (0.2 μm Millipore). The filters were stored frozen at −20°C until DNA extraction with a kit (NucleoSpin Soil, Macherey-Nagel) according to protocol chapter 5.1 (purification of DNA from water). Cells were washed from the filter membrane using buffer SL2 and Buffer SX. For the 16S rRNA gene, we used the 341F and 806R primers (Sundberg *et al.*, 2013), which target the V3-V4 hypervariable loops. For the 18S rRNA gene, the TAReuk454FWD1 and TAReukREV3 primers from Stoeck *et al.* (2010), targeting the V4 hypervariable loops, were used. All samples were barcoded and sequenced together using the 300 bp paired-end read Illumina platform (Illumina MiSeq V3), at a depth of five Mio reads in total, corresponding to an average of ~71 500 reads per sample. Library preparation and sequencing was performed by LGC Genomics (Berlin, Germany). After sequencing, the reads were demultiplexed (using Illumina bcl2fastq 1.8.4), filtering out sequences with more than one mismatch in the barcode, missing barcodes, one-sided barcodes or conflicting barcodes. Finally, sequence adapters and primers were removed.

The sequences were analyzed, and amplicon sequence variants (ASVs) were obtained by processing the resulting raw paired-end reads with R (R Core Team, 2013) package DADA2 v1.16.0 24 following the pipeline described here (http://benjjneb.github.io/dada2/tutorial.html), using the script documented in the supplementary data. The DADA2 pipeline resolves ASVs, instead of OTUs as it resolves single-nucleotide differences and does not contain a clustering step (Callahan *et al.*, 2016). In short, the forward and reverse reads were trimmed and filtered separately. The reads were then filtered using expected error filtering. ASVs were inferred from trimmed, filtered and dereplicated reads and subsequently merged and checked for bimeras (chimeric sequences with exactly two parent sequences). After bimera removal, the taxonomy was assigned with the `assignTaxonomy` function from DADA2, using the SILVA (V132, Quast *et al.*, 2012) as reference database for prokaryotes and the Protist Ribosomal (PR2) Reference database version 4.10.0 (Quast *et al.*, 2012) for eukaryotes. The function implements the RDP Naive Bayesian Classifier algorithm (Wang *et al.*, 2007). The default threshold of 80% bootstrap confidence was applied to retain a taxonomic assignment at a given taxonomic level. Non-target groups were removed after taxonomic assignments, removing eukaryote, mitochondria and chloroplast sequences for prokaryotes, and organelles, bacteria, archaea and metazoan sequences for the eukaryote data set. Sequences that were not assigned at the highest taxonomic level were also removed. The final sequence tables contained 318 prokaryotic and 414 eukaryotic ASVs.

### Statistical analysis

Differences between treatments were tested with analysis of variance (ANOVA) with two fixed factors, grazing with three levels (control, copepodamides and copepods) and nutrients with two levels (ambient and supplemented). Student–Newman–Keuls post-hoc procedure was used to explore differences between groups. The multivariate metabarcoding data were transformed with a variance stabilizing transformation (Anders & Huber, 2010) using the DESeq2 and phyloseq R packages (McMurdie and Holmes, 2013; Love *et al.*, 2014) and community dissimilarities were calculated with the Bray–Curtis dissimilarity and visualized using non-metric multi-dimensional scaling. Statistical analysis of and differences in community composition between treatment clusters were tested for with permANOVA (adonis function, vegan R package, Oksanen **et al.*,* 2022).

## RESULTS

The spring bloom communities were dominated by *Pseudochattonella* sp. (Dicthyochophyceae in Fig. 3) visible as a well-defined peak around 15 μm equivalent spherical diameter in Fig. 4A, B. Ciliates, such as *Mesodinium* sp. were also mainly abundant during the spring campaign. The summer plankton community was dominated by smaller flagellate forms (Fig. 4), and microscopic counts revealed approximately equal proportions of diatoms and dinoflagellates (Fig. 3). Copepods removed some of the larger phytoplankton cells in both experiments (Fig. 4). In addition to the removal of larger cells by copepods, smaller cell sizes increased in both copepod and copepodamide-exposed cultures, in particular in the spring campaign; in contrast, larger cells only decreased in grazed treatments, not in response to copepodamides (Fig. 4 C, D, G, H). Note that the average size of *Pseudochattonella* was slightly smaller in grazed equivalent treatments (14.2 ± 0.1 μm) and copepodamide-exposed treatments (14.3 ± 0.1 μm) than in controls (14.7 ± 0.2 μm) (mean ± SE, *P =* 0.05). Consequently, the decreased average size was not solely the result of size-selective feeding. A comprehensive list of taxa identified by light microscopy is shown in the supplementary data.

**Fig. 2:**
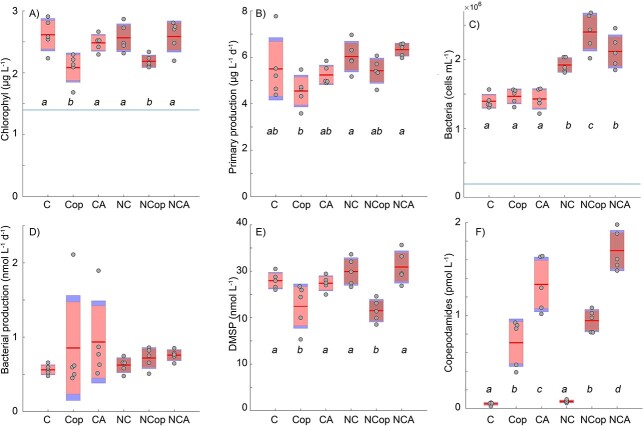
Analysis from summer experiment. Chlorophyll (**A**) was significantly lower in the grazed treatments independent of nutrient additions. The blue horizontal line indicates initial concentration. (**B**) Primary production largely mirrors the chlorophyll content but with a tendency of higher production in enriched flasks. The bacterial counts (**C**) were significantly higher in fertilized treatments, and in particular in the grazed fertilized treatment. (**D**) Bacterial production (thymidine incorporation) was more variable and not significantly different between treatments at the *P =* 0.05 level. (**E**) DMSP largely follow the chlorophyll levels with lower concentrations in grazed treatments. Copepodamide concentrations measured at the end of the experiment (**F**) are significantly elevated in both copepod and copepodamide treatments, and highest in the copepodamide treatments. Axis labels: C denotes control, Cop direct grazing by copepods and CA copepodamide addition. N denotes nutrient addition. Filled circles represent raw data; red box, the 95% confidence interval and blue boxes, the standard deviation, n = 5. Letters denote significance *P =* < 0.05.

**Fig. 3:**
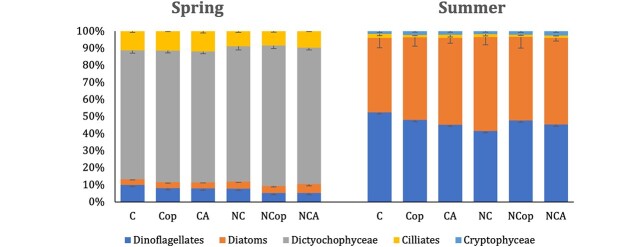
Composition of spring and summer plankton communities based on microscopic counts as percentage of the total. The spring communities were dominated by *Pseudochattonella* sp*.* (Dictyochophyceae) whereas the summer communities were dominated by dinoflagellates and diatoms. For detailed species composition see supplementary data. Values are mean of five replicates and error bars denote standard error of mean. C: control, Cop: direct grazing by copepods, CA: copepodamide addition, and N denotes nutrient addition.

**Fig. 4:**
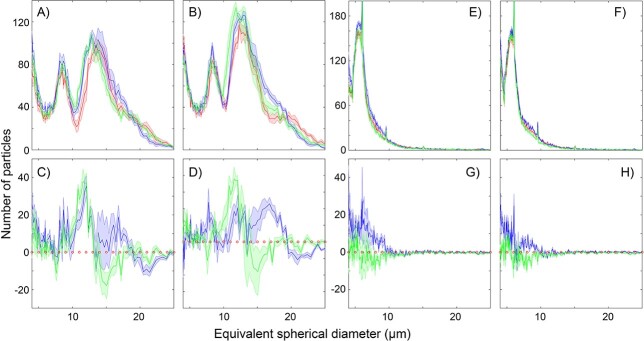
Size distribution of cells determined by Coulter counts. Red lines denote controls, green lines denote copepod-grazed treatments and blue lines denote copepodamide exposed treatments. Panel **A** shows the size distribution in non-fertilized treatments and **B** the fertilized treatments in spring. Panels **E** and **F** show the non-enriched and enriched treatments, respectively, in summer. Bottom panels **C**, **D**, **G** & **H** show the deviation from control (represented by red dotted line) in the copepod and copepodamide treatments from treatments in the panels above. Note that grazers (green) consistently remove larger cells and favour smaller cells. Copepodamides had a similar positive effect on smaller cells but without the reduction of larger cells. Lines denote mean values of five replicates with shaded standard error.

The summer campaign revealed clear effects of both copepod grazing and nutrient addition. Chlorophyll values, used as a proxy for phototrophic biomass, were reduced by 18% by grazers compared to controls (*P <* 0.05, Fig. 2A). The direct grazing effect also manifested in a trend towards lower primary production in copepod treatments, compared to copepodamide and control treatments (Fig. 2B); this trend is either due to grazing or recycling. Grazed treatments had lower concentration of DMSP (*P <* 0.05, Fig. 2E). Bacteria mainly responded to the nutrient enrichment with higher bacterial densities at the end of the experiment and altered community composition in enriched treatments (Fig. 2C). Bacteria also responded to the change in trophic structure and reduced grazing pressure. Grazing enhanced the fertilization effect further and the combination treatment of copepod grazing and nutrient addition had the highest bacterial densities at the end of the experiment (*P <* 0.05, Fig. 2C). Moreover, bacteria increased in all treatments compared to the start value (blue horizontal line in Fig. 2C). Bacterial production at the end of the experiment was, however, not significantly different between treatments (*P >* 0.05; Fig. 2D). Copepodamide concentrations at the end of the experiment were around 1 pM in grazed treatments, and slightly higher, 1.4–1.7 pM in the copepodamide exposed treatments (Fig. 2F).

### Metabarcoding

**Fig. 5:**
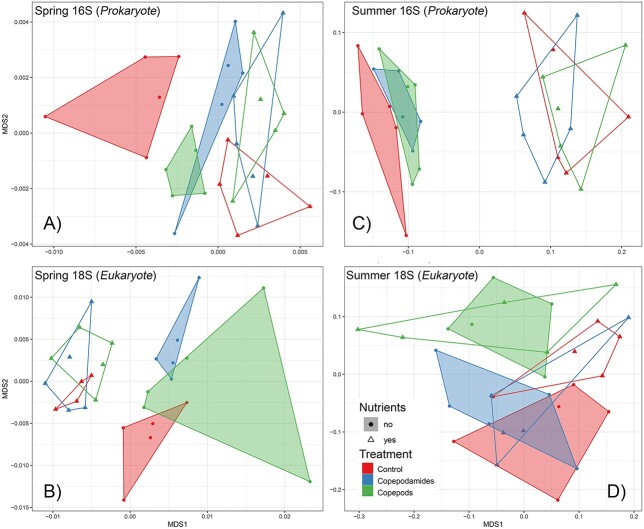
Differences between community composition across treatments (non-metric multidimensional scaling). Each data point represents one community and the distance between data points approximates the multi-dimensional distance in community composition between communities. The distances are calculated as Bray-Curtis dissimilarities based on the ASV composition after applying a variance stabilizing transformation to the raw-count data. The top panel compares prokaryotic communities and the bottom panel eukaryotic communities. The treatments are color coded as follows: red—controls, green—direct grazing by copepods, blue—copepodamides exposure without copepods. Treatments without nutrient addition are represented as circles and treatments with nutrient addition as triangles. Finally, convex hulls demark the spread of the replicates within each of the six treatments, aiding visual comparison: If the convex hulls of two treatments overlap significantly, the communities are similar, if the convex hulls do not overlap, the communities are different. Statistical comparisons of community similarities are presented in the text.

Metabarcoding analyses of eukaryote and prokaryote communities showed a significant change in prokaryote community composition in response to fertilization in summer and spring (*P* < 0.001). The composition of eukaryotes also changed significantly in spring communities (*P* < 0.001) but less so in summer communities (*P* = 0.09). Moreover, the prokaryote composition in grazer and copepodamide-exposed treatments differed from controls in spring (*P =* 0.001) but not summer (*P =* 0.35, Fig. 5). The eukaryote composition in copepodamide and grazer-exposed treatments were different from controls in both spring and summer (*P =* 0.005 and 0.03 respectively). Interestingly, the copepodamide-exposed and grazed treatments were more similar to each other than to controls, and only different for eukaryotes in summer (*P =* 0.002). Thus, the effect of copepods on community structure appears to be driven by a mixture of direct and indirect effects where both direct grazing and copepodamides without grazers affected community structure in the same direction. The effect of grazers and grazer cues was similar in nutrient-enriched experiments but less evident (Fig. 5). In the summer community, three ASVs were significantly different in the copepod treatments compared to the controls. Bacillariophyta, Dinophyceae and Spirotrichea all decreased with the addition of copepods. In the copepodamide treatments the same ASVs for Bacillariophyta decreased and a further two ASVs of Dictyochophyceae and Mamiellophyceae were also significantly reduced when compared the controls (*P* = < 0.05). In the spring community, Litostomatea and Opisthokonta decreased with the addition of copepods. Dinophyceae increased with copepods and copepodamide addition. Full details of multivariate analysis can be found in the supplementary data.

## DISCUSSION

The summative parameters showed a clear effect of copepod grazing (Fig. 2), whereas the addition of copepodamides only affected the size distribution of the communities. In contrast, metabarcoding data analyses revealed that the species composition of the food web changed in a similar way in response to both direct grazing and copepodamide additions in both eukaryote and prokaryote community composition. Thus, indirect structuring effects of grazers and grazer cues are partly masked by functional redundancy, likely contributing to the variable results on trophic cascades in marine plankton (Shurin *et al.*, 2002). Interestingly, the altered community structure observed in both eukaryotes and prokaryotes has a degree of similarity between grazed and copepodamide treatments. This suggests that grazer-induced changes in community structure are partially driven by info-chemicals such as copepodamides rather than direct grazing. There was, however, a trend towards differences between copepodamide-induced and direct-grazed treatments that would likely manifest if longer incubation times were used. The short incubation time of 3 days is sufficient for changes to manifest in fast growing taxa such as procaryotes. In contrast, eukaryotic taxa typically have longer generation times so that longer incubation times in larger mesocosm settings would be more appropriate to investigate this trend further. The difference in community composition is likely driven by the active and physical grazing activity of the copepods and additional indirect effects, such as nutrient regeneration and release of dissolved organic carbon during feeding (Møller *et al.*, 2003). In addition, copepods exude many more compounds other than copepodamides (Selander *et al.*, 2016), and the effect of copepodamides is, consequently, a conservative estimate of indirect effects of copepods. The influence of indirect effects of copepods will depend on the specific traits of favoured taxa. For instance, if cells producing toxic compounds affecting particular components of the food web are favoured, indirect effects may facilitate algal bloom formation, including harmful events (Prevett *et al.*, 2019; Trapp *et al.*, 2021).

The presence of copepods and copepodamides had distinct effects on species composition in the spring bloom community. Clusters overlapped more in the summer but showed the same general pattern as the spring especially for the prokaryote community composition, when copepod densities were higher (Fig. 5). However, the spring bloom community was atypical in the year of the field study (2017) and was dominated by the ichthyotoxic taxon *Pseudochattonella*. Cell size of *Pseudochattonella* decreased in response to both copepods and copepodamides. Reduced cell size has previously been observed in response to copepodamides for both diatoms and dinoflagellates (Grønning and Kiørboe, 2020; Ryderheim *et al.*, 2021). The reasons for the decrease in average cell size in response to grazing pressure are complex. Copepods typically clear larger particles at higher rates than smaller cells (Berggreen *et al.*, 1988), which is mainly driven by inherent higher encounter-rates of larger and faster-swimming prey (Visser and Kiørboe, 2006). Smaller cells thus benefit from reduced encounter rates with copepods, but only marginally (Ryderheim **et al.*,* 2021). Grønning and Kiørboe (2020) have discussed additional reasons for decrease in cell size: one being that defensive metabolites are more concentrated in smaller cells, and the other, only valid for diatoms, is that the frustule is thicker in smaller cells and therefore more robust against grazing (Hamm *et al.*, 2003; Assmy *et al.*, 2013).

The prokaryote community is not directly preyed upon by copepods, instead the changes are the consequence of indirect effects of copepod presence such as nutrient regeneration, trophic cascades driven by consumption, and/or chemical cues from consumers. The addition of nutrients generally weakened the response to copepods or copepodamides, which suggests that part of the effect of the copepods (Fig. 5) may indeed be driven by the regeneration of resources (Møller, 2007). On the other hand, the similarities between the effect of copepods and copepodamides alone (which do not directly cause nutrient regeneration) suggest the involvement of trait-mediated trophic cascades. Copepodamides may also serve as a substrate, favouring some heterotrophic bacteria and contributing to altering community structure through selective nutrient enrichment. Concentration of copepodamides were low, however (in the pM range; Fig. 2), which suggests that cascading effects were more likely and led to the observed structuring effect. The indirect effects of copepodamides may, for example, intimidate microzooplankton ciliates (Arias *et al.*, 2021), leading to predator release of smaller phytoplankton and heterotrophs, such as heterotrophic nanoflagellates, which graze on the prokaryote community. Sign-switching trophic cascades are quite common and are found in a wide variety of ecosystems (Pace *et al.*, 1999).

A longer incubation period may have increased the effects on the community structuring, this experiment lasted for 3 days only with ecologically relevant levels of grazers. In comparison, for example, Trapp *et al.* (2021) showed that the history of copepod-grazing intensity is correlated to the amount of phycotoxins in mussels, with a lag effect of 1–2 months. The positive correlation between the dinoflagellate *Lingulodinium polyedra* (prey) and copepod (predator) densities is associated with a similar time lag (Prevett *et al.*, 2019). Thus, it is likely that longer incubations would have resulted in more accentuated effects on community structure. For future experiments, it would be desirable to test the indirect and direct effects of grazers in continuous or semi-continuous cultures using larger incubation volumes.

In conclusion, we find that the structuring effects of copepods were driven by a combination of direct and indirect effects. The indirect effects from copepodamides mainly affected the changes in community composition with little or no effects on bulk ecosystem functioning. Functional redundancy of copepodamide-induced communities may consequently mask trait-mediated cascades in plankton communities that can only be resolved by high-resolution methods such as barcoding. The consequences of structuring effects on ecosystem functioning depends on the traits of favoured taxa. Traits such as decreased size, toxin production, and silification may alter large-scale processes such as harmful algal bloom formation and carbon export whereas other changes may go largely unnoticed.

## Data Availability

The data underlying this article are available in the article and in its online supplementary material.

## References

[ref1] Anders, S. and Huber, W. (2010) Differential expression analysis for sequence count data. Genome Biol., 11, R106. 10.1186/gb-2010-11-10-r106. 20979621 PMC3218662

[ref2] Arias, A., Selander, E., Saiz, E. and Calbet, A. (2021) Predator chemical cue effects on the diel feeding behaviour of marine Protists. Microb. Ecol., 82, 356–364. 10.1007/s00248-020-01665-9. 33459836

[ref3] Assmy, P., Smetacek, V., Montresor, M., Klaas, C., Henjes, J., Strass, V. H., Arrieta, J. M., Bathmann, U.. et al. (2013) Thick-shelled, grazer-protected diatoms decouple ocean carbon and silicon cycles in the iron-limited Antarctic circumpolar current. Proc. Natl. Acad. Sci. USA, 51, 20633–20638. 10.1073/pnas.1309345110 PMC3870680 24248337

[ref4] Båmstedt, U. (1988) The macrozooplankton community of Kosterfjorden, western Sweden -abundance, biomass, and preliminary data on the life-cycles of dominant species. Sarsia, 73, 107–124. 10.1080/00364827.1988.10420677.

[ref5] Båmstedt, U., Hakanson, J. L., Brennerlarsen, J., Bjornsen, P. K., Geertzhansen, O. and Tiselius, P. (1990) Copepod nutritional condition and pelagic production during autumn in Kosterfjorden, western Sweden. J. Mar. Biol., 104, 197–208. 10.1007/BF01313259.

[ref6] Berggreen, U., Hansen, B. and Kiørboe, T. (1988) Food size spectra, ingestion and growth of the copepod Acartia-Tonsa during development - implications for determination of copepod production. J. Mar. Biol., 99, 341–352. 10.1007/BF02112126.

[ref7] Bergkvist, J., Thor, P., Jakobsen, H. H., Wängberg, S. A. and Selander, E. (2012) Grazer-induced chain length plasticity reduces grazing risk in a marine diatom. Limnol. Oceanogr., 57, 318–324. 10.4319/lo.2012.57.1.0318.

[ref8] Bjærke, O., Jonsson, P. R., Alam, A. and Selander, E. (2015) Is chain length in phytoplankton regulated to evade predation? J. Plankton Res., 37, 1110–1119.

[ref9] Calbet, A. (2001) Mesozooplankton grazing effect on primary production: a global comparative analysis in marine ecosystems. Limnol. Oceanogr., 46, 1824–1830. 10.4319/lo.2001.46.7.1824.

[ref10] Calbet, A. and Landry, M. R. (2004) Phytoplankton growth, microzooplankton grazing, and carbon cycling in marine systems. Limnol. Oceanogr., 49, 51–57. 10.4319/lo.2004.49.1.0051.

[ref11] Calbet, A. and Saiz, E. (2005) The ciliate-copepod link in marine ecosystems. Aquat. Microb. Ecol., 38, 157–167. 10.3354/ame038157.

[ref12] Callahan, B. J., McMurdie, P. J., Rosen, M. J., Han, A. W., Johnson, A. J. A. and Holmes, S. P. (2016) DADA2: high-resolution sample inference from Illumina amplicon data. Nat. Methods, 13, 581–583. 10.1038/nmeth.3869. 27214047 PMC4927377

[ref13] Franchini, F. and Steinke, M. (2016) Protocols for the Quantification of Dimethyl Sulfide (DMS) and Other Volatile Organic Compounds in Aquatic Environments, Hydrocarbon and Lipid Microbiology Protocols. Springer, Berlin, Heidelberg, 161–177, 10.1007/8623_2016_206.

[ref14] Grebner, W., Berglund, E. C., Berggren, F., Eklund, J., Harðadóttir, S., Andersson, M. X. and Selander, E. (2019) Induction of defensive traits in marine plankton—new copepodamide structures. Limnol. Oceanogr., 64, 820–831. 10.1002/lno.11077.

[ref15] Grønning, J. and Kiørboe, T. (2020) Diatom defence: grazer induction and cost of shell-thickening. Funct. Ecol., 34, 1790–1801. 10.1111/1365-2435.13635.

[ref16] Hamm, C. E., Merkel, R., Springer, O., Jurkojc, P., Maier, C., Prechtel, K. and Smetacek, V. (2003) Architecture and material properties of diatom shells provide effective mechanical protection. Nature, 421, 841–843. 10.1038/nature01416. 12594512

[ref17] Kiørboe, T. and Nielsen, T. G. (1994) Regulation of zooplankton biomass and production in a temperate, coastal ecosystem. 1. Copepods. Limnol. Oceanogr., 39, 493–507.

[ref18] Lindström, J., Grebner, W., Rigby, K. and Selander, E. (2017) Effects of predator lipids on dinoflagellate defence mechanisms-increased bioluminescence capacity. Sci. Rep., 7, 13104. 10.1038/s41598-017-13293-4. 29026130 PMC5638803

[ref19] Long, J. D., Smalley, G. W., Barsby, T., Anderson, J. T. and Hay, M. E. (2007) Chemical cues induce consumer-specific defenses in a bloom-forming marine phytoplankton. Proc. Natl. Acad. Sci. USA, 104, 10512–10517. 10.1073/pnas.0611600104. 17563379 PMC1965544

[ref20] Love, M. I., Huber, W. and Anders, S. (2014) Moderated estimation of fold change and dispersion for RNA-seq data with DESeq2. Genome Biol., 15, 550. 10.1186/s13059-014-0550-8. 25516281 PMC4302049

[ref21] Martin, J. L., Santi, I., Pitta, P., John, U. and Gypens, N. (2022) Towards quantitative metabarcoding of eukaryotic plankton: an approach to improve 18S rRNA gene copy number bias. Metabarcoding and Metagenomics, 6, 245–259, e85794. 10.3897/mbmg.6.85794.

[ref22] McMurdie, P. J. and Holmes, S. (2013) Phyloseq: an R package for reproducible interactive analysis and graphics of microbiome census data. PLoS One, 8, 1–11, e61217. 10.1371/journal.pone.0061217. 23630581 PMC3632530

[ref23] Møller, E. F. (2007) Production of dissolved organic carbon by sloppy feeding in the copepods Acartia tonsa, Centropages typicus, and Temora longicornis. Limnol. Oceanogr., 52, 79–84. 10.4319/lo.2007.52.1.0079.

[ref24] Møller, E. F., Thor, P. and Nielsen, T. G. (2003) Production of DOC by Calanus finmarchicus, C. Glacialis and C. Hyperboreus through sloppy feeding and leakage from fecal pellets. Mar. Ecol. Prog. Ser., 262, 185–191.

[ref25] Nielsen, G. Æ. and Bresta, A.-M. (1984) Guidelines for the Measurement of Phytoplankton Primary Production, Baltic Marine Biologists Publication 1, 2nd edn. National Agency of Environmental Protection, Charlottenlund.

[ref26] Nielsen, T. G. and Kiørboe, T. (1994) Regulation of zooplankton biomass and production in a temperate, coastal ecosystem. 2. Ciliates. Limnol. Oceanogr., 39, 508–519. 10.4319/lo.1994.39.3.0508.

[ref27] Oksanen, J., Simpson, G. L., Blanchet, F. G., Kindt, R., Legendre, P., Minchin, P. R., O’Hara, R. B., Solymos, P.. et al. (2022) Vegan: Community Ecology Package, 2.6–2, R Foundation for Statistical Computing, Vienna (Austria).

[ref28] Pace, M. L., Cole, J. J., Carpenter, S. R. and Kitchell, J. F. (1999) Trophic cascades revealed in diverse systems. Trends Ecol. Evol., 15, 483–488. 10.1016/s0169-5347(99)01723-1 10542455

[ref29] Preisser, E. L., Bolnick, D. I. and Benard, M. F. (2005) Scared to death? The effects of intimidation and consumption in predator-prey interactions. Ecology, 86, 501–509. 10.1890/04-0719.

[ref30] Prevett, A., Lindström, J., Xu, J., Karlson, B. and Selander, E. (2019) Grazer-induced bioluminescence gives dinoflagellates a competitive edge. Curr. Biol., 29, 564–565. 10.1016/j.cub.2019.05.019 31211972

[ref31] Quast, C., Pruesse, E., Yilmaz, P., Gerken, J., Schweer, T., Yarza, P., Peplies, J. and Glöckner, F. O. (2012) The SILVA ribosomal RNA gene database project: improved data processing and web-based tools. Nucleic Acids Res., 41, 590–596. 10.1093/nar/gks1219 PMC3531112 23193283

[ref1r] R Core Team (2013). R: A Language and Environment for Statistical Computing. R Foundation for Statistical Computing, Vienna. http://www.R-project.org/.

[ref32] Rigby, K. and Selander, E. (2021) Predatory cues drive colony size reduction in marine diatoms. Ecol. Evol., 11, 11020–11027. 10.1002/ece3.7890. 34429899 PMC8366847

[ref33] Ryderheim, F., Selander, E. and Kiørboe, T. (2021) Predator-induced defence in a dinoflagellate generates benefits without direct costs. ISME, 15, 2107–2116. 10.1038/s41396-021-00908-y. PMC8245491 33580210

[ref34] Ryther, J. and Sanders, J. (1980) Experimental evidence of zooplankton control of the species composition and size distribution of marine phytoplankton. Mar. Ecol. Prog. Ser., 3, 279–283. 10.3354/meps003279.

[ref35] Selander, E., Berglund, E. C., Engström, P., Berggren, F., Eklund, J., Harðardóttir, S., Lundholm, N., Grebner, W.. et al. (2019) Copepods drive large-scale trait-mediated effects in marine plankton. Sci. Adv., 5, eaat5096. 10.1126/sciadv.aat5096. 30801004 PMC6382395

[ref36] Selander, E., Heuschele, J., Nylund, G. M., Pohnert, G., Pavia, H., Bjærke, O., Pender-Healy, L. A., Tiselius, P.. et al. (2016) Solid phase extraction and metabolic profiling of exudates from living copepods. PeerJ, 4, 1–18, e1529. 10.7717/peerj.1529. 26788422 PMC4715450

[ref37] Selander, E., Kubanek, J., Hamberg, M., Andersson, M. X., Cervin, G. and Pavia, H. (2015) Predator lipids induce paralytic shellfish toxins in bloom-forming algae. Proc. Natl. Acad. Sci. USA, 112, 6395–6400. 10.1073/pnas.1420154112. 25918403 PMC4443330

[ref38] Selander, E. S., Jakobsen, H. H., Lombard, F. and Kiørboe, T. (2011) Grazer cues induce stealth behavior in marine dinoflagellates. Proc. Natl. Acad. Sci. USA, 108, 4030–4034. 10.1073/pnas.1011870108. 21368128 PMC3054020

[ref39] Shurin, J. B., Borer, E. T., Seabloom, E. W., Anderson, K., Blanchette, C. A., Broitman, B., Cooper, S. D. and Halpern, B. S. (2002) A cross-ecosystem comparison of the strength of trophic cascades. Ecol. Lett., 5, 785–791. 10.1046/j.1461-0248.2002.00381.x.

[ref40] Stibor, H., Vadstein, O., Diehl, S., Gelzleichter, A., Hansen, T., Hantzsche, F., Katechakis, A., Lippert, B.. et al. (2004) Copepods act as a switch between alternative trophic cascades in marine pelagic food webs. Ecol. Lett., 7, 321–328. 10.1111/j.1461-0248.2004.00580.x.

[ref41] Stoeck, T., Bass, D., Nebel, M., Christen, R., Jones, M. D., Breiner, H. W. and Richards, T. A. (2010) Multiple marker parallel tag environmental DNA sequencing reveals a highly complex eukaryotic community in marine anoxic water. Mol. Ecol., 19, 21–31. 10.1111/j.1365-294X.2009.04480.x. 20331767

[ref42] Strickland, J. D. H. and Parsons, T. R. (1972) A practical handbook for seawater analysis. Fish Res, Board, Canada, Ottawa, 169, 1–311.

[ref43] Strom, S., Wolfe, G., Holmes, J., Stecher, H., Shimeneck, C., Sarah, L. and Moreno, E. (2003) Chemical defense in the microplankton I: feeding and growth rates of heterotrophic protists on the DMS-producing phytoplankter Emiliania huxleyi. Limnol. Oceanogr., 48, 217–229. 10.4319/lo.2003.48.1.0217.

[ref44] Sundberg, C., Al-Soud, W. A., Larsson, M., Alm, E., Yekta, S. S., Svensson, B. H., Sørensen, S. J. and Karlsson, A. (2013) 454 pyrosequencing analyses of bacterial and archaeal richness in 21 full-scale biogas digesters. FEMS Microbiol. Ecol., 85, 612–626. 10.1111/1574-6941.12148. 23678985

[ref45] Suraci, J. P., Clinchy, M., Dill, L. M., Roberts, D. and Zanette, L. Y. (2016) Fear of large carnivores causes a trophic cascade. Nat. Commun., 7, 10698. 10.1038/ncomms10698. 26906881 PMC4766389

[ref46] Tiselius, P. and Kuylenstierna, M. (1996) Growth and decline of a diatom spring bloom: phytoplankton species composition, formation of marine snow and the role of heterotrophic dinoflagellates. J. Plankton Res., 18, 133–155. 10.1093/plankt/18.2.133.

[ref47] Tiselius, P. and Møller, L. F. (2017) Community cascades in a marine pelagic food web controlled by the non-visual apex predator Mnemiopsis leidyi. J. Plankton Res., 39, 271–279.

[ref48] Tollrian, R. and Harvell, C. D. (1999) The Ecology and Evolution of Inducible Defences, Princeton University Press, Princeton.

[ref49] Toth, G. B., Noren, F., Selander, E. and Pavia, H. (2004) Marine dinoflagellates show induced life-history shifts to escape parasite infection in response to water-borne signals. Proc. R. Soc. London, Ser. B, 271, 733–738. 10.1098/rspb.2003.2654 PMC1691648 15209107

[ref50] Trapp, A., Heuschele, J. and Selander, E. (2021) Eavesdropping on plankton—can zooplankton monitoring improve forecasting of biotoxins from harmful algae blooms? Limnol. Oceanogr., 66, 3455–3471. 10.1002/lno.11891.

[ref51] Turner, A. M. and Mittelbach, G. G. (1990) Predator avoidance and community structure-interactions among piscivores, planktivores, and plankton. Ecology, 71, 2241–2254. 10.2307/1938636.

[ref52] Turner, J. T. (2004) The importance of small planktonic copepods and their roles in pelagic marine food webs. Zool. Stud., 43, 255–266.

[ref53] Utermöhl, H. (1931) Neue Wege in der quantitativen Erfassung des planktons. (Mit besonderer Berücksichtigun des Ultraplanktons). Verh. Int. Ver. Theor. Angew. Limnol., 5, 567–596.

[ref54] Van Donk, E., Ianora, A. and Vos, M. (2011) Induced defences in marine and freshwater phytoplankton: a review. Hydrobiologia, 668, 3–19. 10.1007/s10750-010-0395-4.

[ref55] Visser, A. W. and Kiørboe, T. (2006) Plankton motility patterns and encounter rates. Oecologia, 148, 538–546. 10.1007/s00442-006-0385-4. 16586112

[ref56] Wang, Q., Garrity, G. M., Tiedje, J. M. and Cole, J. R. (2007) Naive Bayesian classifier for rapid assignment of rRNA sequences into the new bacterial taxonomy. Appl. Environ. Microbiol., 73, 5261–5267. 10.1128/AEM.00062-07. 17586664 PMC1950982

